# Molecular Dynamics Simulations of Mitochondrial Uncoupling Protein 2

**DOI:** 10.3390/ijms22031214

**Published:** 2021-01-26

**Authors:** Sanja Škulj, Zlatko Brkljača, Jürgen Kreiter, Elena E. Pohl, Mario Vazdar

**Affiliations:** 1 Division of Organic Chemistry and Biochemistry, Ruđer Bošković Institute, Bijenička 54, 10000 Zagreb, Croatia; Sanja.Skulj@irb.hr (S.Š.); Zlatko.Brkljaca@irb.hr (Z.B.); 2 Department of Biomedical Sciences, Institute of Physiology, Pathophysiology and Biophysics, University of Veterinary Medicine, 1210 Vienna, Austria; juergen.kreiter@vetmeduni.ac.at; 3 Institute of Organic Chemistry and Biochemistry, Czech Academy of Sciences, Flemingovo nám. 2, 16610 Prague, Czech Republic

**Keywords:** membrane protein, long-chain fatty acid, proton transfer, purine nucleotide, conductance measurements in model membranes, uncoupling

## Abstract

Molecular dynamics (MD) simulations of uncoupling proteins (UCP), a class of transmembrane proteins relevant for proton transport across inner mitochondrial membranes, represent a complicated task due to the lack of available structural data. In this work, we use a combination of homology modelling and subsequent microsecond molecular dynamics simulations of UCP2 in the DOPC phospholipid bilayer, starting from the structure of the mitochondrial ATP/ADP carrier (ANT) as a template. We show that this protocol leads to a structure that is impermeable to water, in contrast to MD simulations of UCP2 structures based on the experimental NMR structure. We also show that ATP binding in the UCP2 cavity is tight in the homology modelled structure of UCP2 in agreement with experimental observations. Finally, we corroborate our results with conductance measurements in model membranes, which further suggest that the UCP2 structure modeled from ANT protein possesses additional key functional elements, such as a fatty acid-binding site at the R60 region of the protein, directly related to the proton transport mechanism across inner mitochondrial membranes.

## 1. Introduction

Uncoupling protein 2 (UCP2) belongs to the mitochondrial SLC25 superfamily of anion transporters. It was implicated in the pathogenesis of multiple physiological and pathological processes, such as diabetes, ischemia, metabolic disorders, (neuro) inflammation, cancer, and aging. Based on its proton transporting function, UCP2 was first suggested to act as a mild uncoupler to reduce oxidative stress [1,2,3]. Later, it was shown to transport C4 metabolites out of mitochondria [4], facilitating the tricarboxylic acid (TCA) cycle. A recently proposed dual transport function for UCP2 (proton and substrate) increases the similarity of UCP2 to the ANT (also abbreviated as AAC in literature), which transports protons [5,6,7], additionally to ATP/ADP exchange.

The mechanism of how UCP2 controls proton transport across mitochondrial membranes is still not understood. So far, it is established that long-chain fatty acids (FAs) are an integral part of the mechanism and are crucial for proton transfer [8,9,10]. Currently, several mechanistic models exist that explain the proton transfer mechanism. In the first one, so-called the “FA cycling” model, FAs act as protonophores. Due to the excess of protons in the mitochondrial intermembrane space, FA carboxyl anions are easily protonated and they can flip-flop across the membrane very fast in the neutral form to the matrix [11,12,13] where a proton is subsequently released. After that, UCP2 facilitates the otherwise very slow transfer of the negatively charged fatty acid by a still unknown mechanism back to the intermembrane space and the cycle starts again [1,8,14]. The dependence of H^+^ transport rate on FA saturation, FA chain length [9] and fluidity of the membrane [15] indicates that FA^−^ transport likely occurs at the protein−lipid interface.

The second group of models does not involve flip-flop of FAs. Instead, it proposes that carboxyl groups of negatively charged amino acids of the UCPs can accept a proton from a FA and transport it through the hypothetic channel in the UCP (“FA proton buffering” model) [16,17]. Alternatively, the FA anion binds in the cavity inside the UCP interior (“FA shuttle” model). Upon proton binding to the FA anion, a conformational change occurs which shuttles the FA together with a proton, which is subsequently released in the mitochondrion matrix and the cycle is repeated [6,18].

Currently, the consensus on the exact mechanism of how UCP2 works is far from being reached, mainly due to the shortage of reliable structural information. A potential breakthrough in the UCP2 investigation occurred in 2011 when an NMR structure of UCP2 was published [19]. In theory, the structure should have served as an ideal starting point for all potential molecular simulations and detailed structural and mechanistic analyses. Unfortunately, it turned out that the UCP2 structure extracted from commonly used detergent dodecyl phosphocholine (DPC) is not functionally relevant [20]. Moreover, it is now quite established that alkyl phosphocholine detergents destabilize and denature α-helical membrane proteins, leading to a distorted protein secondary structure. It raises important questions on the appropriateness of alkyl phosphocholine detergents as the extraction media for the determination of membrane protein structure by solution NMR. A lively debate is currently still taking place whether the disturbance of the protein structure by these types of detergents is prohibitive for further understanding of the protein function [21,22,23,24] or if it can still be used for capturing the most important functional aspects [25,26,27]. Despite recent developments in membrane protein structure determination, such as detergent-free solubilization of membrane proteins using styrene-maleic acid lipid particles [28,29] and cryo-EM keeping the lipid environment intact [30,31], the handling of small mitochondrial carriers, and in particular uncoupling proteins, remains challenging because of their size and low abundance in mitochondrial membranes. 

Molecular dynamics (MD) simulations are an attractive complementary option for studying membrane proteins, provided that sampling times are sufficiently long to sample their dynamics in membranes adequately [28,29,30]. Membrane proteins are encoded by ca. 30% of the human genome and their total number is predicted to be significantly higher [31]. Only about 1000 unique membrane protein structures are determined today [32] representing a small fraction of the total number of membrane proteins found in humans. However, since an increasing number of membrane protein structures are determined by solution NMR using contentious alkyl phosphonates as the extraction media [33], long MD simulations (in the microsecond time range) in combination with homology modeling [34,35] often represent the only available option for studying membrane protein structure and dynamics. 

Currently, MD simulations of monomeric UCP proteins reported in the literature are primarily based on the available UCP2 NMR structure [20,36]. MD simulations of UCP2 protein made by Zoonens and coworkers indicated that DPC detergent induced large structural deformations of UCP2 protein helices, which in turn created a large water channel, thus facilitating continuous water leakage across the protein [20]. Since the proton conductance is unattainable under these conditions, its experimental measurements have additionally confirmed that UCP2 protein, extracted with the help of DPC detergent, is not functionally relevant. In contrast, the closely related UCP1 protein extracted using DPC and the structurally different detergent TX-100 remained physiologically active [20]. Interestingly, a recent study showing the oligomerization of UCP2 monomers did not describe differences between NMR and homology model structures [37].

Motivated by the lack of relevant MD simulations for the monomeric structures which would help to decipher the function of UCP2 protein in membranes, we turned to homology modeling using the structure of mitochondrial ADP/ATP carrier (ANT, PDB code 1OKC) [38] as a template for UCP2 MD simulations. ANT, a member of the mitochondrial carrier protein family SLC25 [39], is also found in inner mitochondrial membranes. Its primary function is to exchange ADP against ATP across mitochondrial membranes [40,41,42]. However, it has been reported that ANT also works as a proton carrier, similar to UCP proteins, by a mechanism still undetermined at the molecular level [5,6,7]. Taking into account the sequence identity between ANT and UCP2 of 24% [43], a similarity in the overall shape containing six membrane domains, as well as sharing of the proton transporting function in mitochondria, we chose 1OKC structure as a starting template for homology modeling and subsequent microsecond MD simulations. Finally, we compared the simulation results with the data obtained in model membranes reconstituted with UCP2 to validate our MD model.

## 2. Results 

### 2.1. Structural Properties of Modeled Membrane Proteins

**Figure 1:**
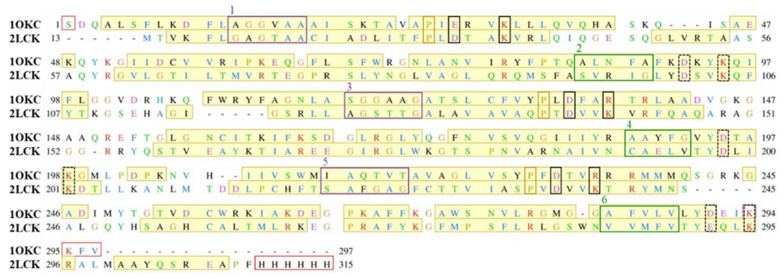
Alignment of the primary sequences of UCP2 protein (PDB code: 2LCK) and bovine ANT protein (PDB code: 1OKC). Secondary structure alpha helices are denoted in the yellow shade, and missing residues from the crystallographic structure are presented with red boxes. Residues that constitute the salt bridge network at the cytoplasmic side are shown in dashed black boxes, while potential residues that could constitute the salt bridge network at the matrix side are enclosed in solid black boxes. πGπxπG (helices 1, 3 and 5) and πxxxπ (helices 2, 4 and 6) motifs are depicted in violet and green boxes, respectively. Residues responsible for proline kinks are enclosed in yellow boxes.

Firstly, we aligned the primary sequences of two proteins, murine UCP2 protein and bovine ANT protein (Figure 1). Although the homology of both proteins is 24%, the crucial amino acid positions and motifs characteristic for even and odd-numbered transmembrane helices, namely πGπxπG (helices 1, 3 and 5) and πxxxπ (helices 2, 4 and 6), are conserved. Furthermore, the positions of amino acids corresponding to matrix or cytosolic salt bridge network, as well as proline kinks at odd-numbered helices, also remain preserved (Figure 1).

Taking into account that strategic pillars, including cytosolic and matrix salt bridges as well as shape-forming proline kinks in both structures were conserved, we felt that it was safe to take the ANT structure as a starting point for subsequent MD simulations. In our previous MD simulations of two different crystallographic structures belonging to differently open ANT states towards the cytosolic [38] or matrix [42] side of the inner mitochondrial membrane, we have shown that it is possible to capture important conformational changes of the protein embedded in the membrane within microsecond MD simulations [44]. Moreover, we have also revealed that an order shorter timescales of ca. 200 ns, used in previous MD simulations of UCP2 NMR structures in DOPC bilayers [20], were not sufficiently long for a more relevant description of critical regions in the protein, such as reversible salt bridge breaking and forming [44]. In addition to MD simulations of the UCP2_h_, we also repeated simulations of UCP2_NMR_ based on the NMR structure of UCP2 [20] and compared them to referent ANT simulations at relevant microsecond timescales. A schematic representation of the UCP2 structure is depicted in Figure S1.

**Figure 2:**
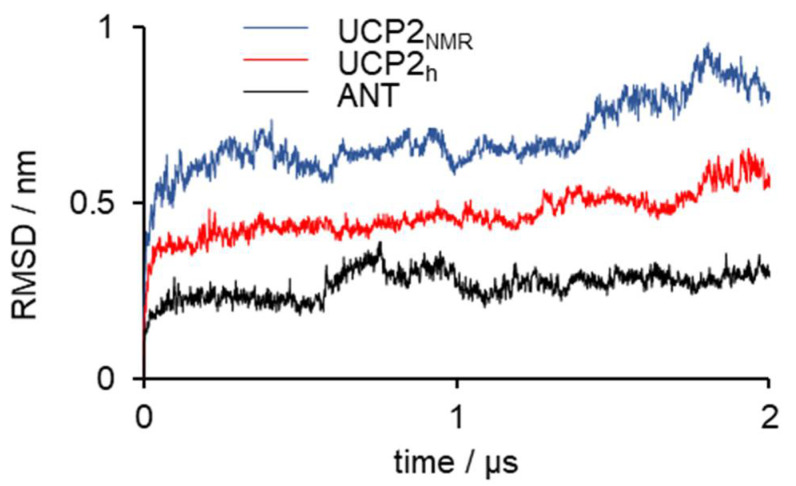
Time propagation of the RMSD values for UCP2_NMR_, UCP2_h_ and the referent structure ANT. The RMSD is calculated for backbone carbon atoms (C_α_) of protein.

**Figure 3:**
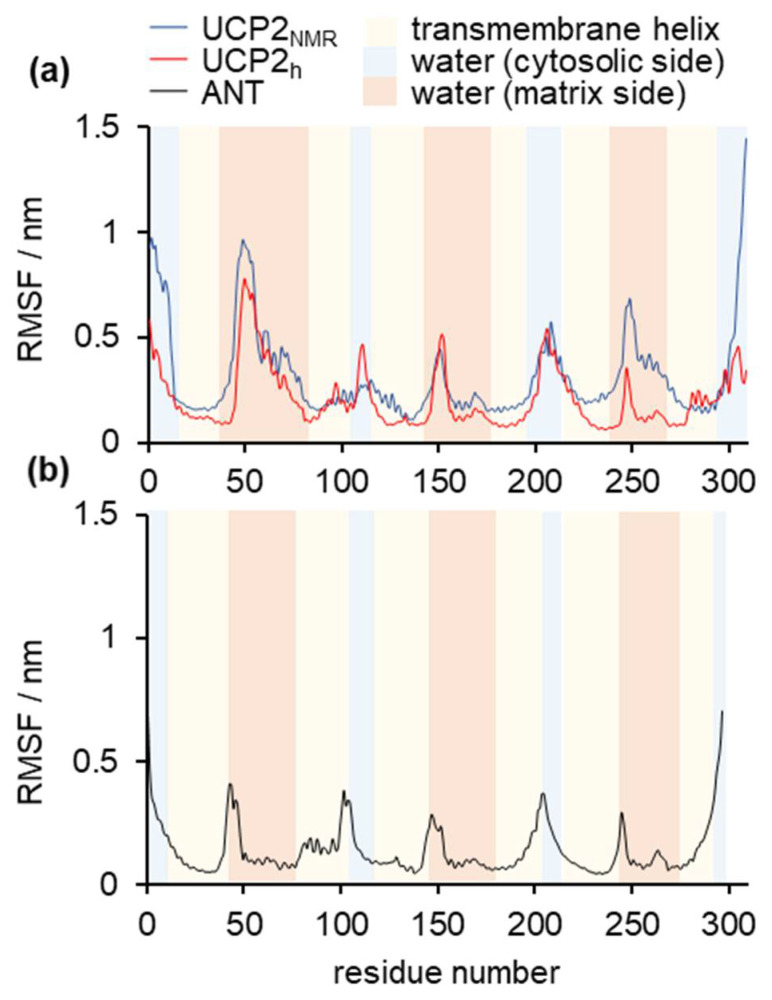
RMSF analysis of (**a**) UCP2_NMR_ and UCP2_h_ structures and (**b**) referent ANT structure. The RMSF is calculated for backbone carbon atoms (C_α_) of protein. Light red color corresponds to protein residues inside the phospholipid bilayer, light blue represents protein residues immersed in water at the cytosolic side, whereas dark pink corresponds to the protein residues immersed in water at the matrix side.

Figure 2 shows the time evolution of root mean square deviation (RMSD), which indicates the three studied protein structures’ stability in time. RMSD deviation was the largest for UCP2_NMR_, which was not surprising, given the fact that this structure obtained in alkylphosphocholine detergent was determined in a non-optimal environment [20,22,33]. It was visible that the extension of simulations by Zoonens et al. [20] showed even larger deformations of the structure (especially after 1 μs), which will be analyzed in more detail in later sections. MD simulations of UCP2_h_ were more stable, although a small increase in the RMSD occurred at the end of simulation time. However, these instabilities were not as severe as in the UCP2_NMR_ simulations and were of a similar order of magnitude as the RMSD oscillations observed for the referent ANT structure. However, although very useful for the general description of protein stability, the RMSD analysis was a simple “one-number” analysis and did not contain information on the conformational changes of specific residues [45]. For this reason, we turned to root mean square fluctuation (RMSF) analysis (Figure 3), which provided important (but not time-resolved) data on the flexibility of particular residues. Importantly, we saw that the UCP2_NMR_ structure was more flexible (and less stable) around residues found in the water phase, oriented to the matrix side (especially around residues 250–270 and C-terminus) compared to UCP2_h_ and referent ANT structures. Figure S2 shows the time evolution of the UCP2_NMR_ and UCP2_h_ secondary structures which remained preserved for both structures in the simulation time.

**Figure 4:**
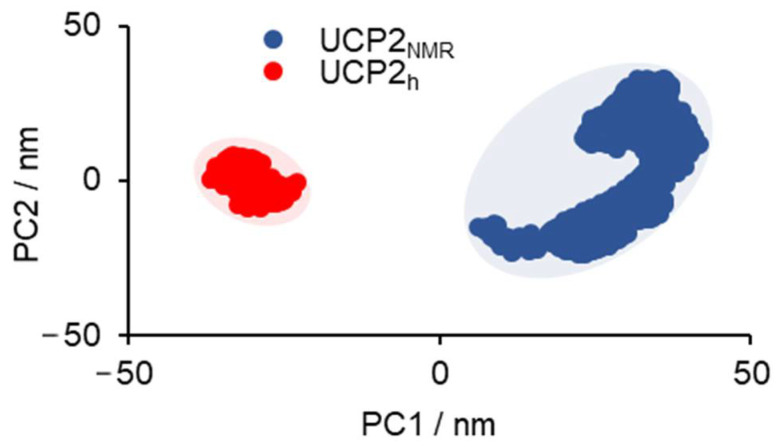
PCA analysis-2D projection of UCP2_NMR_ and UCP2_h_ protein conformations onto common first and second principal components (PC1 and PC2) are presented in blue and red color, respectively.

Finally, another very useful analysis of general structural parameters was obtained by the principal component analysis (PCA) of backbone carbon atoms of the protein. PCA is a procedure that reduces a multidimensional complex set of all possible conformational degrees of freedom to lower dimensions along which the main conformational changes of protein are identified. The PCA analysis of UCP2_NMR_ and UCP2_h_ is shown in the Figure 4. We can see that the area spanned by the first two principal components (PC1 and PC2) was much larger in the case of UCP2_NMR_ structure in comparison to UCP2_h_ structure. This further supports the above analysis, showing that UCP2_NMR_ structure was more flexible and less stable in DOPC phospholipid bilayer. The analysis indicates that the protein structure tried to find its optimal position and a proper fold in the membrane, which was not attainable at a microsecond time scale and probably orders of magnitude longer simulation times were needed. In contrast, the area spanned by PC1/PC2 in the UCP2_h_ structure was much smaller and relatively compact, demonstrating that it was stable in the bilayer within our simulation time. It was in line with RMSD and RMSF analyses and with reported microsecond MD simulations of ANT protein [44].

### 2.2. Stability of Salt Bridges Exposed to the Cytosolic and Matrix Side of the Inner Mitochondrial Membrane

As a next step, we now focused on the stability of the salt bridge networks formed at the cytosolic and matrix sides of the modeled UCP2 structure (Figure S1). Opening and closing the cytosolic and matrix side of the ANT protein via salt bridges, which are connected to the transport of ADP and ATP nucleotides across inner mitochondrial membranes, involves at least 10 kcal mol^−1^ for breaking the salt bridge network [40,41,42]. However, it is essential that water does not leak through the protein interior since it would abolish strictly controlled proton transport due to water-mediated ion exchange, as had been shown by functional leakage assays [20]. Therefore, as an initial prerequisite for controlled proton transfer, the UCP2 protein should be impermeable to water in order not to allow short-circuiting of the system, which is possible only if the salt bridge network is closed and constricts the protein at the matrix [38] or cytosolic side [42] as found in the corresponding crystallographic structures of ANT and subsequent MD simulations [44]. These experimental results further indicated that the proton transport mechanism, either via UCPs or ANT [6], was not controlled by direct transport of proton through the protein interior, but involved the transport of FA anion (and in turn proton) alongside the protein/lipid interface [1,8,9,46]. 

**Figure 5:**
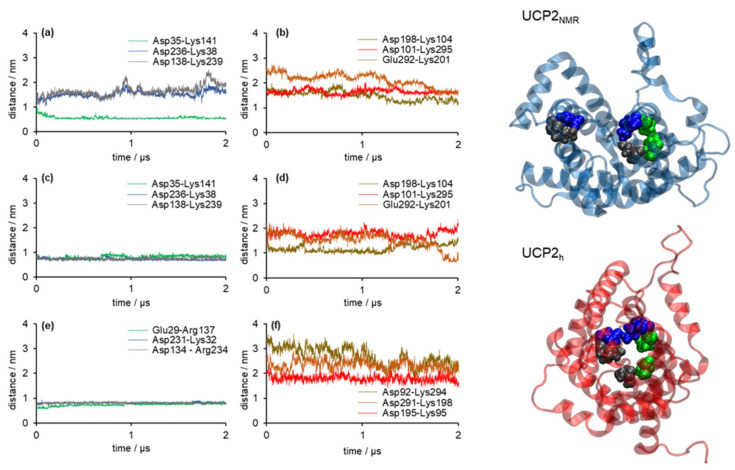
Analysis of the salt bridge network for UCP2 model based on (**a**,**b**) the UCP2 NMR structure (UCP2_NMR_), (**c**,**d**) the UCP2 model based on the crystallographic structure of ANT (UCP2_h_), and (**e**,**f**) the referent ANT structure. Distances between residues that can form a salt bridge network at the matrix side are shown in panels (**a**,**c**,**e**). Distances between residues that can form a salt bridge network at the cytoplasmic side are shown in panels (**b**,**d**,**f**). Distances are calculated between centers of mass of the corresponding residues. A top-down view on the matrix exposed side of selected protein snapshots of UCP2_NMR_ and UCP2_h_ structures is shown on the right.

The salt bridge networks analysis showed that in the case of the UCP2_NMR_ structure (Figure 5a,b), only one residue pair (Asp35-Lys141), located at the matrix side of the protein, permanently formed a salt bridge within our simulation time. In contrast, two other salt bridges located at the matrix side (Asp236-Lys38 and Asp138-Lys239) were not making a salt bridge, as well as three other salt bridge pairs at the cytosolic side (Asp198-Lys104, Asp101-Lys295, and Glu292-Lys201). On the other hand, salt bridges at the matrix side formed in the case of UCP2_h_ structure (Asp35-Lys141, Asp236-Lys38, and Asp138-Lys239) were stable and persistent (Figure 5c) just as in the case of the analogous salt bridges in the referent ANT structure (Glu29-Arg137, Asp231-Lys32, Asp134-Arg234) presented in Figure 5e. Cytosolic salt bridges were partially closed in UCP2_h_ (Figure 5d). In contrast, they were fully opened in the case of ANT (Figure 5f). These results imply that water leakage should be largely suppressed in the case of the UCP2_h_ structure due to the closed matrix side of the protein, which is the pivotal condition for the protein structure to have a relevant functional role in the proton transfer mechanism. We reached similar conclusions by analysis of the referent ANT structure. However, in the case of UCP2_NMR_ structure, we showed that due to the opened matrix side of the protein, water leakage was possible across the protein interior (more details are found in the next section). It was similar to the observations by Zoonens et al. from their shorter analogous MD simulations [20]. We should also mention that in the case of UCP2_h_ and ANT structures, the distances between pairs of negatively charged residues at the matrix side (i.e., EG-motif), which were highly conserved across mitochondrial ADP/ATP carriers [47], kept three-fold pseudosymmetry in contrast to the UCP2_NMR_ structure where this motif was not conserved, and distances between the negatively charged residues were larger (Figure S3). In this way, we further showed that the UCP2_NMR_ structure was unstable and functionally irrelevant when embedded in phospholipid bilayers, which were structurally significantly different compared to the alkyl phosphocholine environment serving as an extracting agent [20,22,33].

### 2.3. Water Leakage across the Protein and Permeability Calculations

**Figure 6:**
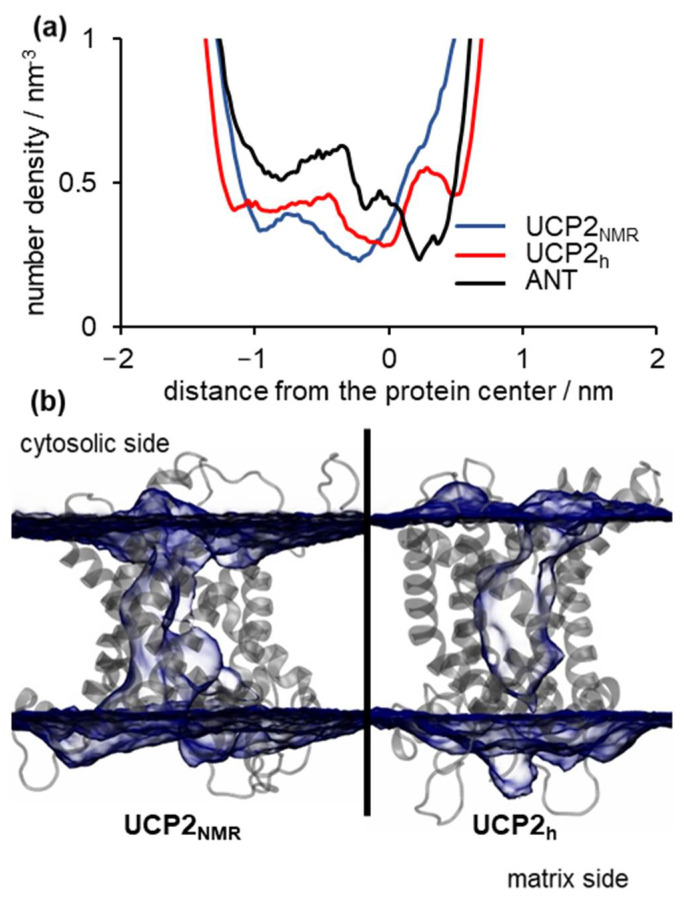
(**a**) *z*-averaged water number density based on 2 μs simulations for UCP2_h_, UCP2_NMR_ and referent ANT structures, (**b**) snapshots presenting volume map of water in transparent blue with surface isovalue set to 0.2 for UCP2_NMR_ structure (left side) and homology modeled UCP2_h_ structure (right side). Cytosolic and matrix sides of UCP2 protein are indicated.

The analysis of the salt bridge networks in the previous section suggests that UCP2_NMR_ structure should be more water permeable due to the simultaneously open matrix and cytosolic sides of the protein in contrast to the partially closed UCP2_h_ structure (Figure 5). To quantitatively analyze this assumption, we performed a detailed analysis of the water density inside the protein for both structures and calculated corresponding water osmotic permeability coefficients *P_f_* using the method described in Zoonens et al. [20]. The analysis of averaged water density inside the protein along the *z*-axis for UCP2_NMR_, UCP2_h_ and the referent ANT structure is shown in Figure 6a, with the time evolution shown in Figure S4. Interestingly, although the averaged number density of water was averaged across *z*-coordinate and did not include the differences in the *x*- and *y*- directions, the minimal value of the number density was similar for all structures, being less than a half of the water molecule per nm^3^. Thus, it was not very informative of the possible formation of a continuous water channel, which would possibly enable water-mediated direct proton transfer leading to the inactive UCP protein [44]. However, we should mention here that the presence of a continuous water channel is not a key prerequisite for efficient proton transfer across the membrane protein and that electrostatic effects resulting in a high energy barrier for proton transfer predominate, such as in a case of aquaporins [48,49]. 

A better look at Figure 6a revealed that the area, and in turn the total volume of water, was largest in the case of the UCP2_NMR_ structure (blue curve), in contrast to UCP2_h_ and ANT number density profiles, which were considerably wider (red and black curve, respectively). This is better visualized in Figure 6b, where we saw that the average volume map of water was continuous along the protein interior (left panel) in the case of UCP2_NMR_ structure. In contrast, two disjointed volume maps existed in the case of the UCP2_h_ structure, indicating that the water channel was not formed (right panel), similar to ANT protein.

However, these analyses are still not fully quantitative, and therefore we turned to water osmotic permeability calculations *P_f_*, to compare the data to other systems. The results of the calculations are shown in Table 1.

Osmotic permeability coefficients were calculated for four different membrane structures. First, we calculated water osmotic permeability for the equilibrated UCP2_NMR_ structure (i.e., only after short initial equilibration), which closely corresponded to the experimental NMR structure. The calculated *P_f_* is (5.7 ± 0.4) × 10^−13^ cm^3^ s^−1^, which is comparable to the value of 5.3 × 10^−13^ cm^3^ s^−1^ obtained by Zoonens et al. for an analogous system [20]. Similarly to their observations, the *P_f_* decreased after 200 ns to (3.2 ± 0.2) × 10^−13^ cm^3^ s^−1^) and finally after 2 μs it assumed the value of (1.3 ± 0.1) × 10^−13^ cm^3^ s^−1^ which showed a certain collapse of the water pore in the protein. However, this number was still comparable to the water osmotic permeability of the α-hemolysine, where this value was calculated to be 1.9 × 10^−12^ cm^3^ s^−1^ [50]. Since these values were comparable, it was clear that the UCP2_NMR_ structure was behaving quite similarly to the water channel, which was physiologically irrelevant for UCP2 function [20]. On the other hand, the water permeability coefficient of UCP2_h_ structure, calculated after 2 μs, was by three orders of magnitude lower being *P_f_* = (2.0 ± 0.5) × 10^−16^ cm^3^ s^−1^. This was additionally confirmed by simple counting of water molecules that crossed across the protein, where we saw that UCP2_h_ and ANT protein were virtually impermeable in contrast to the UCP2_NMR_ structure (Table S1). These results are in accordance with the general structure analysis described in the previous sections, and promote UCP2_h_ structure as a potentially relevant structure for further MD simulation and mechanistic studies. We should mention here that the MD simulations of UCP2 based on ANT homology structure presented in Reference [37] agreed with the presented MD simulations. In particular, the constriction at the matrix side and opening of the cytosolic side of UCP2 protein had been observed as well, together with the low number density of water inside the protein cavity similar to the UCP2_h_ structure (Figure 5 and Figure 6).

### 2.4. Binding of ATP in the UCP2 Cavity

**Figure 7:**
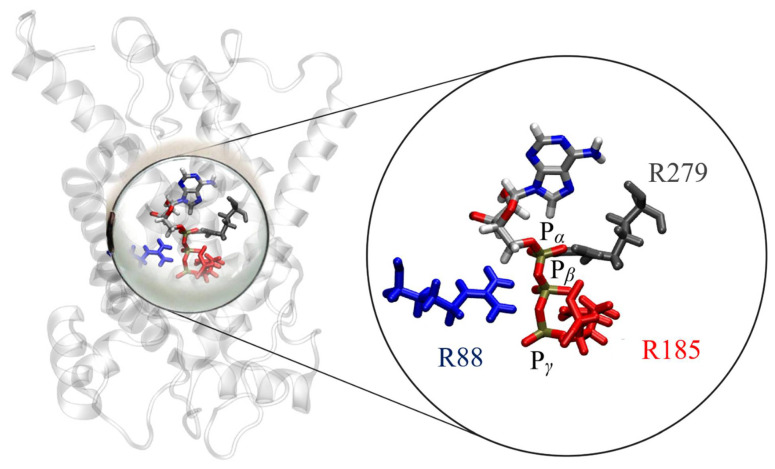
Simultaneous ATP nucleotide binding to three arginines (R88, R185, R279) in the case of UCP2_h_ protein, with R279 (depicted in gray) being found to primarily bind to P_α_. R88 (shown in blue) binds to P_β_ (occasionally to P_α_), while R185 (depicted in red) binds predominantly to P_γ_. Water molecules are omitted for the sake of clarity.

To further verify key functional elements of the UCP2_h_ structure, we performed a series of MD simulations (see Simulation Details) to evaluate the binding properties of ATP nucleotide in the UCP2 cavity which are known to inhibit proton transport in UCPs. It has been suggested in the literature that three positively charged arginine residues in the UCP1 cavity bind a negatively charged phosphate group, which leads to the conformational change of the protein and inhibition of proton transport [2,51,52,53]. This mechanism can be extended to other UCP proteins as well since the arginine residues responsible for nucleotide binding are conserved in other homologs as illustrated in Figure S5. MD simulations of ATP binding in the UCP2_h_ cavity show that the ATP phosphate group binds tightly in the protein cavity, having all three phosphate groups bound to arginines R88, R185, and R279 (Figure 7). This can be inspected by analyzing average distances between phosphorous atoms present in ATP and the center of mass of arginine residues in the UCP2 structure (Figure S6). In particular, we observed that in the case of UCP2_h_ structure, the binding of phosphate to arginine residues was tight, with all three phosphate groups bound to arginine residues R88, R185 and R279. This was in striking contrast to UCP2_NMR_ structures after 20 ns and also after 2 μs where simultaneous binding of ATP phosphate groups to arginine residues did not occur and average distances between the groups were significantly larger (Figure S6), implying in turn weaker binding of ATP. This is in line with previously suggested binding motifs of ATP in UCP1 and UCP3 [54] and very tight binding of GDP in the UCP1 cavity determined by titration calorimetry experiments [52]. Moreover, it has been found that in the case of AAC3 protein, binding of carboxyatractyloside (CATR) inhibitor at the analogous location in the protein cavity as ATP was by several orders of magnitude weaker if the AAC protein structure was obtained by extraction with DPC detergent in contrast to the native crystallographic structure [24]. Therefore, we believe that the molecular description of ATP binding in the UCP2_h_ cavity further promotes the relevance of the homology modelled structure for further MD simulation studies. 

### 2.5. Binding of Fatty Acid to UCP2

In previous sections, we focused on the general structural parameters of the UCP2 protein obtained by MD simulations. Finally, we turned to additional experimental verification of MD results by complementary experiments using model membranes to prove whether the suggested UCP2_h_ structure might be physiologically relevant.

**Figure 8:**
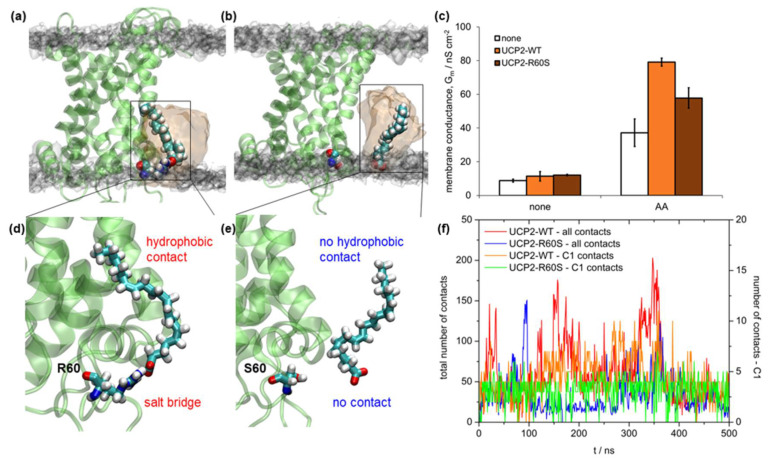
Snapshots of 500 ns simulations of UCP2 homology modeled structures (UCP2_h_) with added arachidonic acid anion (AA^−^). A starting structure of protein used for binding calculations is taken from UCP2h MD simulations after 2 μs. UCP2_h_ structure with AA^−^ (**a**) bound to R60 and (**b**) not bound to the mutated binding site S60. A volume map of phosphorus atoms of DOPC lipid is presented in transparent gray color (surface isovalue set to 0.0064) and volume map of AA^−^ is presented in transparent orange color (surface isovalue set to 0.02). (**c**) Specific membrane conductance of lipid bilayers in the absence of protein (white), and presence of UCP2-WT (orange) or UCP2-R60S (brown). Membranes were made of 45:45:10 mol.% DOPC:DOPE:CL reconstituted with 15 mol.% AA where indicated. Lipid and protein concentrations were 1.5 mg/mL and 4 µg per mg of lipid, respectively. The buffer solution contained 50 mM Na_2_SO_4_, 10 mM TRIS, 10 mM MES and 0.6 mM EGTA at pH = 7.34 and *T* = 306 K. Data are represented as the mean and standard deviation from three independent experiments. (**d**) Zoomed region of AA^−^ binding to UCP2_h_ structure. (**e**) Zoomed region of AA^−^ binding to UCP2-R60S mutant structure. (**f**) Total number of contacts within 0.35 nm between all AA^−^ atoms and UCP2-WT and (red color) and UCP2-R60S mutant structures (blue color), respectively. Total number of contacts within 0.35 nm between AA^−^ carboxyl atom towards UCP2_h_ (orange color) and UCP2-R60S structures (green color).

NMR titration experiments, as well as proton flux assay measurements, suggest that a patch of positively charged residues around R60 residue in UCP2 (consisted of K271, R267, R40, and R71 residues) is relevant for the proton transport mechanism since it serves as a binding site for FA anion [25]. We should stress here that the proton flux assay measurements presented in Reference [25] were performed in liposomes with correctly folded protein. It suggests that the active site in UCP2 is preserved regardless of the protein extraction medium. We performed experiments with the recombinant mutant UCP2 to check whether the mutation from R60 to S60 affected the proton conductance (see details in Materials and Methods). We also performed 500 ns of complementary MD simulations with the AA anion (AA^−^) to visualize the binding process at the molecular level. Figure 8c shows the comparison of the experimental total membrane conductance (*G_m_*) in two systems: the wild type UCP2-WT protein and the UCP2-R60S mutant protein. Both proteins were measured in the presence and absence of AA. We observed two effects. Firstly, the addition of AA^−^ was essential for the increase of *G_m_* as shown in our previous works [9,10], as the conductance increased by order of magnitude compared to the neat WT protein. Secondly, the mutation from R60 to S60 had a significant effect on the *G_m_*, thus further supporting the hypothesis that R60 is a possible binding site for AA^−^. It agreed with NMR titration experiments and proton flux assays [25]. Moreover, the addition of ATP, which is an efficient inhibitor of the UCP2 proton-transporting function [3,10,43,55], also showed that it was effective only in the UCP2-WT and less effective in the UCP2-R60S (Figure S7), confirming that R60 had an important role in the proton transport mechanism. However, based on the present MD simulation results, it is still questionable whether other positively charged residues along the outer protein ring at the matrix side could also serve as potential binding sites of AA^−^.

A detailed analysis of MD simulations was in full agreement with conductance measurements using model membranes. In Figure 8a,d (which is a zoomed region of the binding site) we can see that AA^−^ fitted nicely to the binding site, at the same time having two distinct modes of interaction, i.e., salt bridge formation between R60 and AA^−^ and stabilizing hydrophobic interactions between AA^−^ and protein α-helices. This was also clearly visible in the analysis of total contacts between AA^−^ and protein (Figure 8f, red and orange curves). Conversely, interaction between AA^−^ and S60 was severely diminished (Figure 8b,e), resulting in no formation of a salt bridge between S60 and AA^−^, as well as a greatly reduced number of total contacts between AA^−^ and protein α-helices within 0.35 nm (blue and green curve, Figure 8c). Interestingly, the number of contacts within 0.35 nm between carboxylic carbon atom C1 of AA^−^ was similar in both cases. The present analysis implies that the unsaturated 20:4 AA anion, with its four cis double bonds, which are conformationally quite restricted, fits much better to the UCP2_h_-WT structure than the UCP2-R60S mutant. 

Interestingly, the activation of UCP2-WT with saturated 20:0 arachidic acid (ArA) showed lower *G_m_* than with its unsaturated 20:4 counterpart [10], thus further pointing out the importance of hydrophobic contacts for proper binding of FA anion to R60, which were reduced and entropically unfavored in a far more flexible fully saturated ArA. 

**Figure 9:**
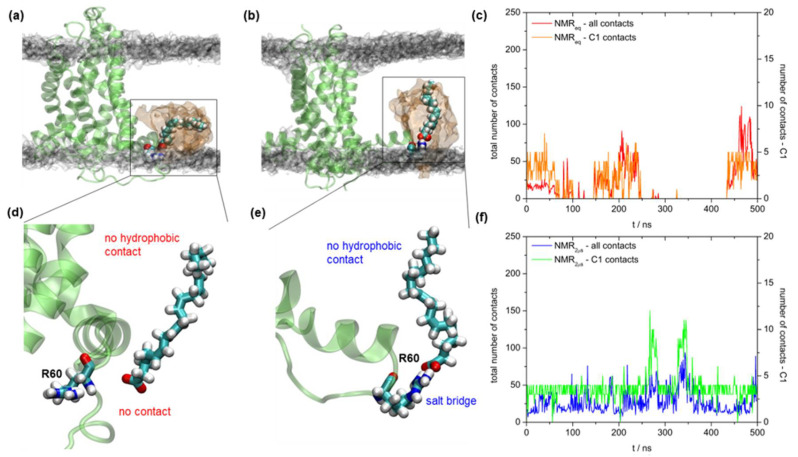
Snapshots of 500 ns simulations of UCP2_NMR_ structures with added arachidonic acid anion (AA^−^). The starting structures of protein used for binding calculations are taken from UCP2_NMR_ MD simulations (**a**) after equilibration and (**b**) after 2 μs. A volume map of phosphorus atoms of DOPC lipid is presented in transparent gray color (surface isovalue set to 0.0064) and volume map of AA^−^ is presented in transparent orange color (surface isovalue set to 0.02). (**c**) Total number of contacts within 0.35 nm between all AA^−^ atoms and UCP2_NMR_ after equilibration. (**d**) Zoomed region of AA^−^ binding to UCP2_NMR_ structure after equilibration. (**e**) Zoomed region of AA^−^ binding to UCP2_NMR_ structure after 2 μs. (**f**) Total number of contacts within 0.35 nm between all AA^−^ atoms and UCP2_NMR_ after 2 μs.

Finally, we also analyzed how AA^−^ binds to the UCP2_NMR_ structure in two cases, after equilibration (to mimic the NMR experimental structure) and after 2 μs of MD simulations (to see the effect of relaxation in the bilayer). In the case of the equilibrated NMR structure, AA^−^ bound very poorly to R60, showing no permanent salt bridge formation (evidenced by a smaller number of contacts between C1 atom and protein in comparison to the UCP2_h_ structure shown in Figure 8) and very few total contacts with the protein (Figure 9a,c,d). In addition to the increased permeability of the protein to water, which is a fundamental structural problem, we also saw that binding of AA^−^ to R60 was not established, which even further disqualified UCP2_NMR_ structure in regard to new mechanistic studies. After 2 μs of MD simulations, the total number of contacts remained low and the situation was actually even worse as AA^−^ could not achieve proper hydrophobic interactions with one of the α-helices which was displaced from the rest of the UCP2 protein structure (Figure 9b,e,f). This implies a possible denaturation or even disintegration of the experimental UCP2_NMR_ structure when transferred from the alkyl phosphonate detergent environment to the phospholipid bilayer milieu. 

## 3. Discussion

The absence of the relevant UCP structure hinders the understanding of UCP2 transport mechanisms and biological functions. To find a possible remedy for the current situation, we turned to homology modeling using ANT crystallographic structure and experimental UCP2 NMR structure as starting structures for long microsecond MD simulations of UCP2 proteins (UCP2_h_ and UCP2_NMR_). We showed by microsecond MD simulations that UCP2_h_ structure is almost water impermeable (*P_f_* = 2.0 × 10^−16^ cm^3^ s^−1^), with a water osmotic permeability coefficient lower by three orders of magnitude than the corresponding UCP2_NMR_ structure after 2 μs of MD simulation time (*P_f_* = 1.3 × 10^−13^ cm^3^ s^−1^). This is a consequence of the salt bridge network formation at the matrix side of the protein in UCP2_h_, formed by arginine/lysine and aspartate/glutamate residues, as well as conservation of the threefold symmetry between negatively charged residues at the matrix side (EG motif), similar to water-impermeable ANT proteins [44,56,57]. In contrast, the stable formation of the identical salt bridges and the EG motif at the matrix side of UCP2_NMR_ structure were not observed, thus enabling water to almost freely diffuse across the protein. Finally, we showed that ATP binds to all three arginine residues in the UCP2 cavity only in the case of UCP2_h_ structure, in agreement with previous experimental observations [53,54]. We should mention here that MD simulations of UCP2 proteins are not fully converged (which is almost impossible with the current computational power) and that UCP2_h_ structure is possibly not the global minimum. Still, we believe that the presented UCP2_h_ structure is relevant for future studies since it corresponds well to available experimental data and that its behavior is described sufficiently well.

The described UCP2_h_ structure can be easily transferred to membranes formed by DOPC, DOPE and cardiolipin, which would then more realistically describe the lipid composition of inner mitochondrial membranes and our model membranes. However, at this stage, we believe that further complication of a model system is not necessary, as we have shown previously in MD simulations of ANT proteins in different, more complex environments [44]. Due to a high complexity of membrane protein/lipid bilayer systems and a necessity for exceedingly long simulation times, pitfalls resulting from incomplete coverage of whole phase space in heterogeneous systems, even at the microsecond timescales and relatively small bilayers, are most likely. 

To test the validity of MD simulation results regarding UCP2 functionality, we compared the binding of UCP2 activator AA to both models, UCP2_h_ and UCP2_NMR_. Additionally, we measured the activation of the recombinant UCP2 due to the AA binding. A larger membrane conductance of the UCP2 in the presence of AA compared to the UCP2-R60S, in which the putative AA^−^ binding site was modified, is in full agreement with MD simulation results. It convincingly shows that the suggested AA^−^ binding region around R60 is available only in the UCP2_h_ protein structure and that in UCP2-R60S the binding of AA^−^ is prevented. This shows that the UCP2_h_ structure correctly predicts the binding of AA^−^ and is sensitive to selective mutations, such as R60S. In contrast, the similar binding of FA to the UCP2_NMR_ structure, both the experimental one and after 2 μs of MD simulation time is not optimal, thus further questioning the stability and physiological relevance of experimental NMR structure.

The proposed MD model of UCP2_h_ can largely contribute to the investigation of the UCP2-mediated proton transport mechanism in general and the protein binding site for FA in particular. Currently, two basic mechanisms are proposed for UCP1-mediated proton transport (see Introduction). A crucial difference between the “FA cycling” and “FA shuttle” hypotheses is a localization of the binding site for the activating FA. The “FA shuttle” model states that FA^−^ binds inside the pore from the cytosolic side of UCP1 and transfers protons by shuttling from the cytosolic to the matrix side of mitochondria [58]. In contrast, the “FA cycling” hypothesis suggests that FA^−^ binds to protein from the matrix side and is transported to the intermembrane space in its deprotonated form. Our initial results that show that FA can bind from the matrix site contradict the results of patch-clamp experiments on mitoplasts, showing the inability of UCP1 to bind FAs on the matrix side [18]. Further developments of the UCP2 model will pave the way towards clarifying this question and the complete transport mechanism of UCP2.

In conclusion, we propose the protocol involving homology modelling of UCP2 protein based on ANT structure and subsequent microsecond molecular dynamics simulations. It yields a functionally relevant structure, which can be used for future mechanistic studies of proton/FA transfer in mitochondria.

## 4. Materials and Methods

### 4.1. Simulation Details

To analyze the UCP2 protein structure, schematically represented in Figure S1, we simulated and compared two different structures of UCP2 protein and bovine ANT protein as a reference. The simulated structures include (a) the published NMR structure of mUCP2 (PDB code 2LCK, organism *Mus musculus*) [19] determined by NMR molecular fragment replacement, (b) the homology modeled structure of the primary sequence of UCP2 protein to crystallographic structure of ANT protein (PDB code 1OKC, 2.2 Å, high resolution, organism *Bos taurus*) [38] whose homology to mUCP2 is 24%, and (c) a crystallographic structure of ANT protein, which served as a template for the homology modeling of UCP2. Moreover, we used it as a reference structure for investigated UCP2 structures. 

Three different systems were prepared using the membrane builder module of CHARMM-GUI (http://www.charmm-gui.org/) [59,60,61] from three UCP2/ANT starting protein structures immersed in 1,2-dioleoyl-sn-glycero-3-phosphocholine (DOPC) membrane containing 230 lipid molecules, 28,750 water molecules, and 15/19 chloride ions to neutralize the net charge of UCP2/ANT protein. DOPC was selected as it represents one of the main lipids of inner mitochondrial membranes. The homology modeled structure of UCP2 and missing residues (compared to the crystallographically determined structures of ANT)—residue 1 and residues 294–297 for 1OKC and residues 1–13 for 2LCK—were added using USCF Chimera program [62]. All arginines and lysines were prepared in their protonated forms, histidines and cysteines in their neutral forms, with glutamates and aspartates being in their deprotonated forms. CHARMM-GUI membrane builder minimization and equilibration procedure was used for all systems [59]. After equilibration, we simulated each system for 2.0 µs using unbiased all-atom molecular dynamics (MD) simulations in a periodic rectangular box of 9.5 nm × 9.5 nm × 13.5 nm with a time step of 2 fs with CHARMM36m force field [63] and TIP3P water model [64]. 

In order to check the potential binding site of the fatty acid anion in UCP2, we also simulated an additional four systems of UCP2 protein structures with added arachidonic acid anion (AA^−^). AA was used since it shows a substantial effect on the proton conductance across mitochondrial membranes [10]. AA^−^ was added to the binding site arginine R60 in three systems: (a) the homology modelled structure of UCP2 (UCP2_h_) simulated after 2 µs, (b) the NMR structure after initial equilibration which closely resembles experimental NMR structure, and (c) the NMR experimental structure (UCP2_NMR_) simulated after 2 µs. In the fourth system, AA^−^ was added to the mutated binding site (R60 to S60) in UCP2_h_ simulated after 2 µs (UCP2-R60_h_). Each of the four AA^−^ containing systems mentioned above was simulated for 500 ns and analyzed further. 

All production MD simulations were performed in the isobaric-isothermal ensemble (NPT) at *T* = 310 K, which was maintained via Nosé−Hoover thermostat [65,66] independently for three groups: DOPC, water/ions, and protein subsystem with a coupling constant of 1.0 ps^−1^. The pressure was set to 1.013 bar and controlled with semi-isotropic Parrinello−Rahman barostat [67] with a time constant for pressure coupling of 5 ps^−1^. Periodic boundary conditions (PBC) were imposed in all three directions, with long range electrostatic interactions calculated by the particle-mesh Ewald (PME) method [68] with real space Coulomb interactions cut-off at 1.2 nm using a Fourier spacing of 0.12 nm and Verlet cut-off scheme. All simulations were propagated with GROMACS 2018.4 software package [69] and visualized with VMD (Visualize Molecular Dynamics) program [70]. 

### 4.2. Homology Modeling

For the homology modeled structure, we used a sequence from 2LCK protein. Using Blast protein comparative structure modelling [71] in UniProt Protein Knowledgebase [72], we obtained the best matching tertiary structures: 1OKC, 2C3E, and 4C9G. The best matching tertiary structure 1OKC (lowest significance value—4.7 × 10^−20^ and highest alignment score—88) serves as a structure template. From the target-template sequence alignment, we generated 10 models and chose the model with the lowest discrete optimized protein energy (DOPE) score of 0.85 was chosen as the representative structure to carry out simulations. Homology models were constructed using programs MODELLER 9 [73] and Chimera 1.13.1 [62].

### 4.3. Permeability Calculations 

We calculated osmotic permeabilities for four distinct UCP2 structures obtained from unbiased MD simulations, closely following the procedure illustrated by Zoonens et al. [20] and based on the algorithm described by Aksementiev and Schulten [50]. In this respect, the osmotic permeability of homologically modeled UCP2 was calculated using structures obtained after 2 μs of unbiased MD simulation, while the same property was determined for three distinct UCP2 NMR structures, i.e., structures obtained immediately after equilibration, after 0.2 μs and 2 μs of unbiased MD simulation. The four chosen systems were propagated in the NVT ensemble, where position restraints were applied on C_α_ atoms of the protein (500 kcal mol^−1^nm^−2^). The simulations were propagated for 10 ns with a 2 fs time step, with the last 5 ns used in the subsequent analysis. The simulation snapshots were saved every 250 steps, i.e., every 0.5 ps. PBC conditions were applied in all three directions and treated the long-range electrostatics using the PME method (see above for details).

The pore formed in the structure of both UCP2_NMR_ and UCP2_h_ possesses a complex topology; thus, careful choice of the region for the osmotic permeability calculations is necessary. Following the procedure of Zoonens et al. [20], we calculated the permeability taking into account only the central region of the pore. This region was rather well-preserved during simulations, and its topology relatively simple, i.e., it could be accurately described as being roughly cylindrical in nature. In this respect, the chosen region for the UCP2_NMR_ structures was defined by two roughly coplanar rings, each consisting of six C_α_ atoms. Each C_α_ atom was chosen to belong to a different transmembrane helix present in the protein. The chosen C_α_ atoms form a bottom and a top ring, and belong to residues 34, 85, 137, 181, 239, and 274, and residues 20, 101, 120, 194, 227, 288, respectively. Due to a different topology of the UCP2_h_ protein, we used a similar yet somewhat different choice of C_α_ atoms to delineate its central pore. In this respect, a bottom and a top ring were described using C_α_ atoms belonging to residues 34, 82, 137, 181, and 274, and residues 20, 101, 120, 192 and 288, respectively (see Figure S8).

Thus, the region encapsulated between the two chosen rings has the form of a cylinder with bases at the centers of mass of the bottom (R_0_) and the top ring (R_1_), respectively. The axis of this cylinder lies along the vector R_1_–R_0_. The radius of the cylinder (*r* = 2 nm) was chosen so that it is large enough to enclose all water molecules found in the analyzed pore of UCP2 protein.

Water molecules collective displacement within the protein pore at time *t* + ∆*t* of the MD simulation trajectory can now be calculated using the approach developed by Zhu et al. [74], i.e., via

$$n\left(t+\Delta t\right)=n\left(t\right)+\underset{i\in S\left(t,t+\Delta t\right)}{\sum }\left(\frac{\Delta r_{i}\Delta e}{L}\right)$$

where the union of all subsets of water molecules that are found inside the cylinder at time *t* and *t +* ∆*t* is denoted by *S(t +* Δ*t*), Δ**r***_i_* represents the displacement of *i*-th water molecule in the time window *t* to *t +* ∆*t*, **e** represents the unit vector along R_1_–R_0_. *L* denotes the length of a cylindrical region and is approximately equal to 1.95 nm in all considered cases. Importantly, displacements of water molecules that enter or exit the cylindrical region between the two consecutive frames were cut at the boundaries of the region in such a way that only the displacement of such water molecules inside the region is taken into account.

The collective diffusion coefficient of water inside the protein, *D_n_*, was calculated using ⟨*n(t)*⟩^2^ = 2*D_n_t*, with the average being obtained over 100 subtrajectories, each being 50 ps in length (5 ns of overall post-equilibration simulation time/100), Figure S9. The osmotic permeability was estimated using *P_f_* = *v_w_D_n_*, where the average volume of a single water molecule is denoted by *v_w_*. Finally, thus obtained osmotic permeabilities were scaled by a factor of 1/2.87, since real water possesses larger viscosity compared to the used TIP3P water model [50].

### 4.4. Binding of ATP in the UCP2 Cavity

To inspect the geometry of the binding site of ATP in the UCP2 protein cavity, we performed a set of simulations (50 per inspected UCP2 structure). We initially placed the ATP molecule inside the UCP2 cavity for three distinct protein structures, UCP2_h_, and two UCP2_NMR_ structures. More precisely, to represent UCP2_h_, we chose the structure obtained after 2 μs of its respective free MD simulation, while two distinct UCP2_NMR_ structures, namely the structures obtained after 20 ns and after 2 μs of their respective free MD simulation, were utilized to inspect the behavior of ATP in the cavity of UCP2_NMR_. For each UCP2 structure, 50 different MD simulations in the duration of 20 ns each were performed (overall 1 μs per UCP2 structure). The ATP starting position was maintained for each of the 50 simulations, with the initial velocities being randomly generated, following the Boltzmann distribution. Thus, while the starting structure in each 50 simulation sets (for each investigated UCP2 structure) is represented by the same point in the conformational phase space, its position in the momentum phase space is different, representing overall distinct starting structures. To inspect whether the initial conditions biased the obtained results, we performed additional simulations (again 20 ns each) for the UCP2_h_ structure, where we placed ATP molecule in five different spots in the cavity of UCP2_h_ and performed 10 simulations for each starting configuration. In all simulations mentioned above, only the last 10 ns were used in the analysis. The first 10 ns were omitted (equilibration time). CHARMM36m force field parameters were used to describe ATP moiety together with the aforementioned parameters of UCP2 and DOPC lipids. The simulations were performed in the NVT ensemble, with all other MD simulation parameters being identical to the ones applied in the long 2 μs simulations (see Simulation Details).

### 4.5. Chemicals

The 1,2-dioleoyl-sn-glycero-3-phosphocholine(DOPC), 1,2-dioleoyl-sn-glycero-3-phosphoetha-nolamine (DOPE), cardiolipin (CL) from bovine heart, arachidonic acid (AA), Triton X-114 octylpolyoxyethylene, dithiothreitol (DTT), bovine serum albumin (BSA), adenosine and guanosine triphosphate (ATP and GTP), sodium sulfate (Na_2_SO_4_), diammonium hydrogen phosphate ((NH_4_)_2_HPO_4_), 2-(N-morpholino)ethanesulfonic acid (MES), 2-Amino-2-(hydroxymethyl)propane-1,3-diol (Tris), ethylene glycol-bis(β-aminoethyl ether)-N,N,N′,N′-tetraacetic acid (EGTA), hexane, hexadecane and sodium dodecyl sulfate (SDS) were obtained from Sigma-Aldrich (Munich, Germany). Chloroform was from Merck KGaA (Darmstadt, Germany).

### 4.6. Cloning, Mutation and Expression of mUCP2 and Reconstitution into Liposomes

Mouse UCP2 (mUCP2) was cloned and expressed, as described previously [75,76]. In brief, the ORF of mUCP2 was inserted into the pET24a- expression plasmid. For expression of the protein the plasmid of wild type mUCP2 was transferred into *E. coli* cells (strain Rosetta) and grown to reach OD_600nm_ between 0.3 and 0.5. The protein expression was then induced by adding 1 mM isopropyl-β-D-thiogalactopyranoside (IPTG). *E. coli* cells were incubated for 3 h before harvesting by centrifugation. Inclusion bodies (IB) containing the expressed proteins were collected by disruption of cells using a French press following centrifugation at 14,000× *g* [77]. 

In vitro site-directed mutagenesis was carried out on expression plasmids containing the cDNA of mUCP2 as templates. The mutation was introduced with a designed oligonucleotide to alter the R60 (CGT) to S (AGT) using Q5 site-directed mutagenesis kit (New England Biolabs GmbH, Frankfurt am Main, Germany) and confirmed by sequencing. 

Recombinant mUCP2WT and mUCP2R60S were purified and refolded from inclusion bodies, and reconstituted into liposomes according to the previously described protocol [54]. In brief, mUCP3 were solubilized from IB using 2% sarcosyl and 1 mM DTT. 100 mg *E. coli* polar lipid (Avanti polar lipids, Alabaster, AL, USA), 300 μg Triton X-114, 75 μg octyl-polyoxyethylene and 2 mM GTP were added to the solubilized mUCP2. Sarcosyl and GTP were removed by dialysis with the assay buffer (50 mM Na_2_SO_4_, 10 mM Tris, 10 mM MES and 0.6 mM EGTA, pH 7.34). The sample was passed through a hydroxyapatite column (Bio-Rad, Laboratories, Inc., Feldkirchen, Germany) to remove decomposed proteins. Nonionic detergents were eliminated using Bio Beads (Bio-Rad, Germany). The purity of the recombinant proteins was verified by silver staining (Figure S10). The correct folding was proved by the activity assay—protein activation or inhibition.

### 4.7. Measurements of Electrical Parameters of Membranes Reconstituted with mUCP2

Planar lipid bilayers were formed from (proteo-) liposomes [78,79] made of 45:45:10 mol.% DOPC:DOPE:CL. Lipid concentration was 1.5 mg/mL and protein to lipid ratio—4 µg per mg of lipid. Arachidonic acid (AA) at a concentration of 15 mol.% was directly added to the lipid phase before membrane formation. Buffer contained 50 mM Na_2_SO_4_, 10 mM Tris, 10 mM MES and 0.6 mM EGTA at pH = 7.34 and *T* = 306 K. Proper membrane formation was verified by measuring specific capacitance (C = 718 ± 34 nF/cm^2^) that was independent of protein, AA and ATP content. Current−voltage (I-U) measurements were performed with a patch-clamp amplifier (EPC 10 USB, HEKA Elektronik Dr Schulze GmbH, Lambrecht, Germany). Total membrane conductance at 0 mV was obtained from the slope of a linear fit of experimental data at applied voltages from −50 mV to +50 mV (Figure S7). ATP was dissolved in a buffer solution to a concentration of 400 mM and the stock solution pH was adjusted to 7.34. The volume of 3.75 µL of the stock solution was added to 750 µL buffer solution for a final concentration of 2 mM ATP. Incubation time was 30 min at *T* = 306 K. Data were analyzed using Sigma Plot (Systat Software GmbH, Erkrath, Germany). 

## Figures and Tables

**Table 1:** Water osmotic permeability coefficients calculated for four distinct membrane protein structures in different simulation times.

Structure	Permeability (cm^3^ s^−1^)
UCP2_NMR_ after equilibration	(5.7 ± 0.4) × 10^−13^
UCP2_NMR_ after 200 ns	(3.2 ± 0.2) × 10^−13^
UCP2_NMR_ after 2 μs	(1.3 ± 0.1) × 10^−13^
UCP2_h_ after 2 μs	(2.0 ± 0.5) × 10^−16^

## Data Availability

The datasets generated and/or analyzed during this study are available from the corresponding authors on reasonable request.

## Supplementary Materials

The following are available online at https://www.mdpi.com/1422-0067/22/3/1214/s1. Figure S1: A schematic representation of UCP2 protein in the inner mitochondrial membrane, Figure S2: Time propagation of the secondary structure in simulations of UCP2 protein, Figure S3: Analysis of the EG motif in UCP2 and ANT structures, Figure S4: Time evolution of *z*-averaged water number density in simulations of UCP2 protein, Figure S5: Alignment of the primary sequences of UCP1, UCP2 and UCP3 proteins, Figure S6: Analysis of ATP binding in UCP2_h_, UCP2_NMR_ and ANT structures, Figure S7: Experimental measurements of UCP2 conductance, Figure S8: Cylindrical region of UCP2 used for permeability calculations, Figure S9: Mean square displacements (MSDs) for diffusion coefficient calculations, Figure S10: Representative silverstaining of murine UCP2WT and UCP2R60S, Table S1: Number of water molecules passing through the membrane for UCP2_h_, UCP2_NMR_ and ANT.
