# Supplementary material for: Molecular Dynamics Simulations of Mitochondrial Uncoupling Protein 2
Source: Int J Mol Sci. 2021 Jan 26;22(3):1214. doi: 10.3390/ijms22031214 (PMC7866055; doi:10.3390/ijms22031214)
Supplement: Supplementary file 1 [file ijms-22-01214-s001.pdf]

Supporting Information

Molecular Dynamics Simulations of Mitochondrial Uncoupling Protein 2

Sanja Škulj[1], Zlatko Brkljača[1], Jürgen Kreiter[2], Elena E. Pohl[2,*], Mario Vazdar[1,3,*]

[1]Division of Organic Chemistry and Biochemistry, Ruđer Bošković Institute, Bijenička 54, Zagreb 10 000, Croatia

[2]Institute of Physiology, Pathophysiology and Biophysics, Department of Biomedical Sciences, University of Veterinary Medicine, Vienna, Austria

[3]Institute of Organic Chemistry and Biochemistry, Czech Academy of Sciences, Flemingovo nám. 2, Prague 6 16610, Czech Republic

*e-mails: mario.vazdar@irb.hr, elena.pohl@vetmeduni.ac.at

Table of contents:

S-2: Figure S1 A schematic representation of UCP2 protein in the inner mitochondrial membrane

S-3: Figure S2 Time propagation of the secondary structure in simulations of UCP2 protein

S-4: Figure S3 Analysis of the EG motif in UCP2 and ANT structures

S-5: Figure S4 Time evolution of *z*-averaged water number density in simulations of UCP2 protein

S-6: Figure S5 Alignment of the primary sequences of UCP1, UCP2 and UCP3 proteins

S-7: Figure S6 Analysis of ATP binding in $UCP2_h$, $UCP2_{NMR}$ and ANT structures

S-8: Figure S7 Experimental measurements of UCP2 conductance

S-9: Figure S8 Cylindrical region of UCP2 used for permeability calculations

S-10: Figure S9 Mean square displacements (MSDs) for diffusion coefficient calculations

S-11: Figure S10 Representative silverstaining of murine UCP2WT and UCP2R60S

S-12: Table S1 Number of water molecules passing through the membrane for $UCP2_h$, $UCP2_{NMR}$ and ANT

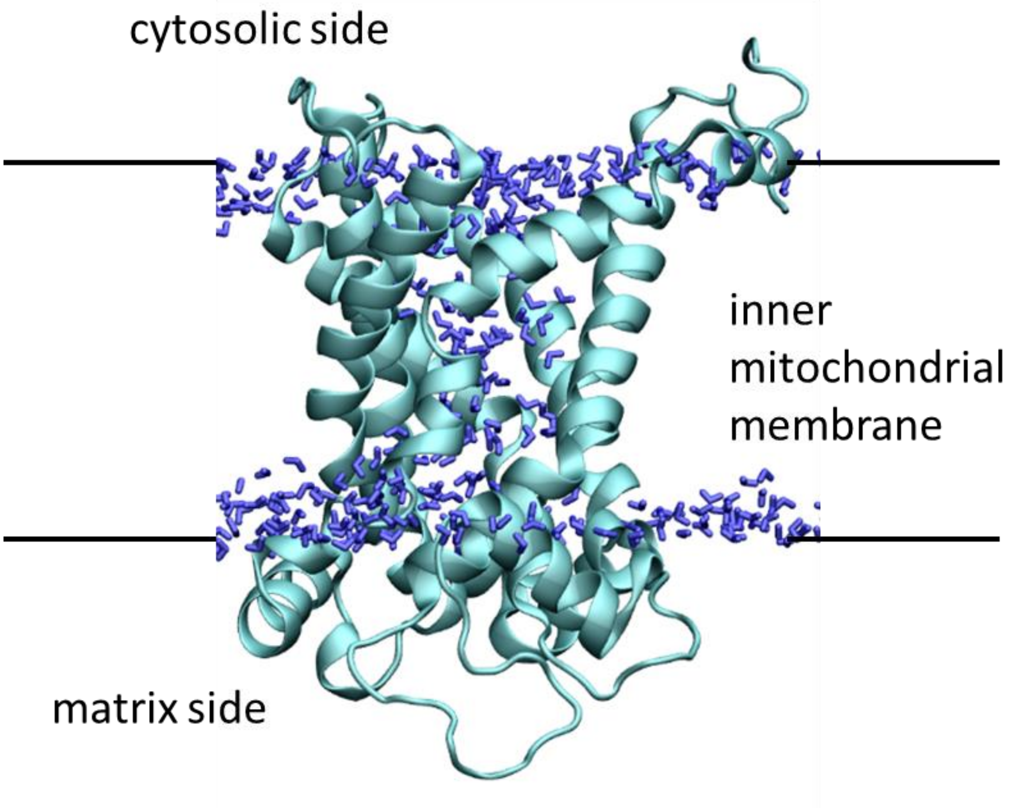

Figure S1. A schematic representation of UCP2 protein in the inner mitochondrial membrane. Six transmembrane alpha-helices are indicated in mint; water is shown in blue color. N- and C-termini are directed towards intermembrane (cytosolic) side.

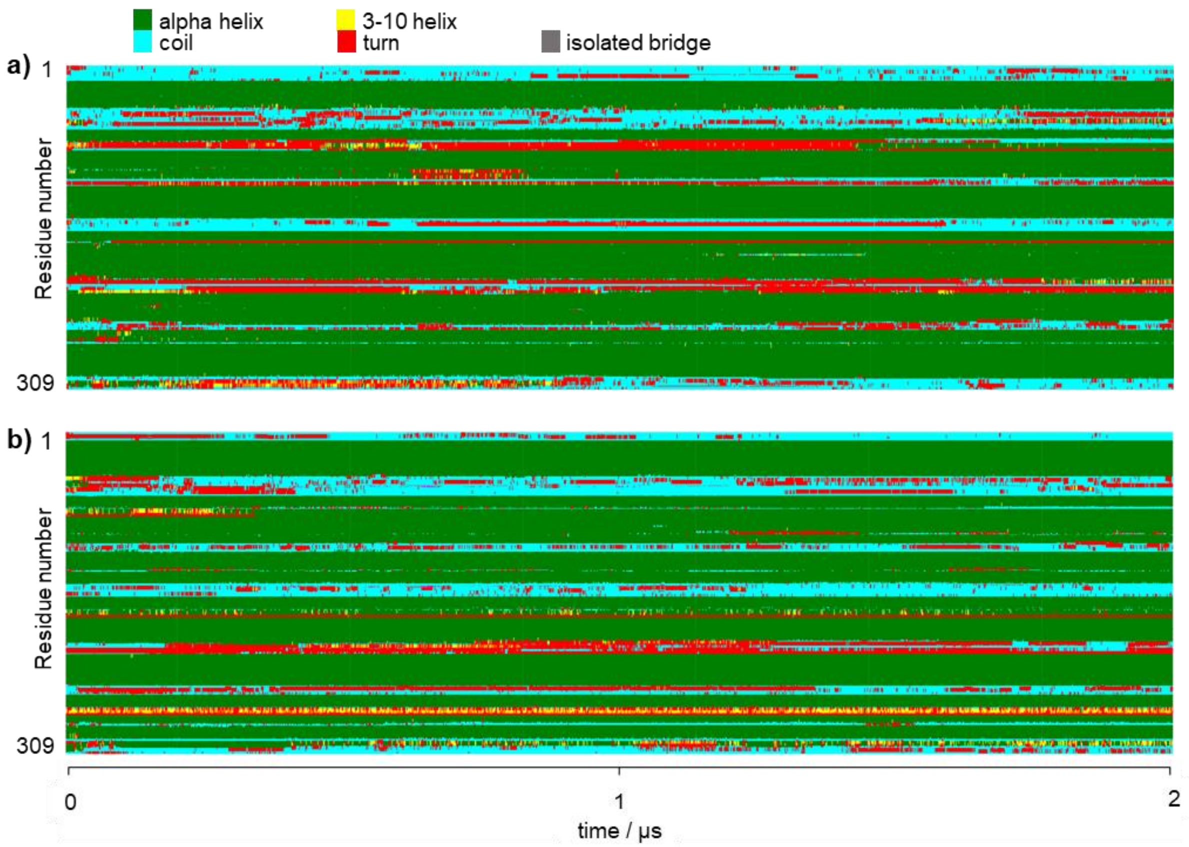

Figure S2. Time propagation of the secondary structure for simulation of (a) $UCP2_{NMR}$ and (b) $UCP2_h$ structures.

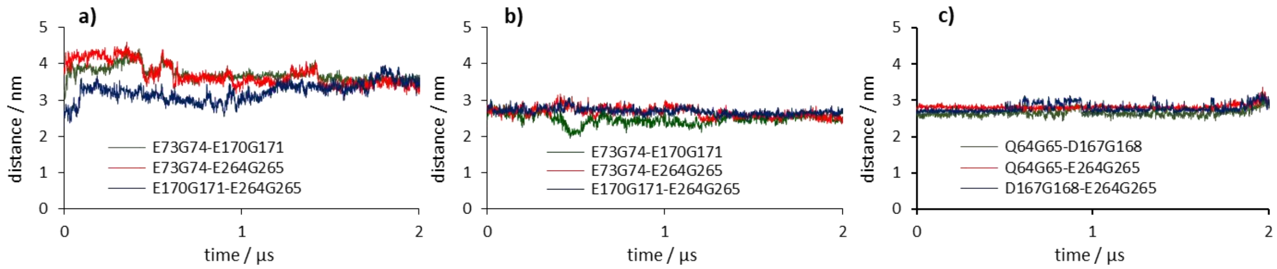

Figure S3. Analysis of the EG motif in: a) $UCP2_{NMR}$, (b) $UCP2_h$ and (c) ANT structures during simulation time.

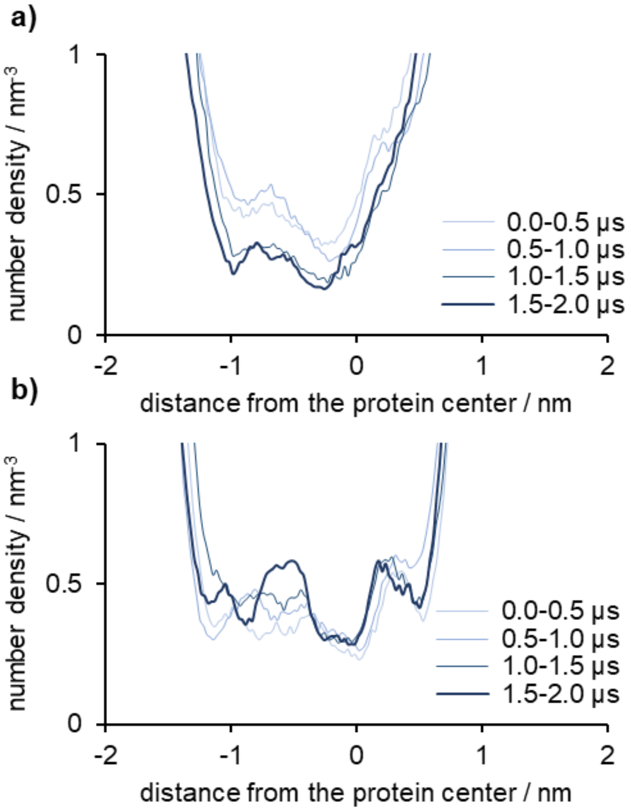

Figure S4. Time evolution of *z*-averaged water number density during 2 μs simulations for (a) $UCP2_{NMR}$ and (b) $UCP2_{h}$ structures.

```
UCP1    1   MVNPTTSEVQPTMGVKIFSAGVSACLADIITFPLDTAKVRLQIQGEGQA----SSTIRYK    56
UCP2    1   MVGFKATDVPPTATVKFLGAGTAACIADLITFPLDTAKVRLQIQGESQGLVRTAASAQYR    60
UCP3    1   MVGLQPSEVPPTTVVKFLGAGTAACFADLLTFPLDTAKVRLQIQGENPG----AQSVQYR    56
            **.   ::* **  **::.**.:**:**::****************. .    : : :*:

UCP1   57   GVLGTITTLAKTEGLPKLYSGLPAGIQRQISFASLRIGLYDSVQEYFSSGRETPASLGNK   116
UCP2   61   GVLGTILTMVRTEGPRSLYNGLVAGLQRQMSFASVRIGLYDSVKQFYT-KGSEHAGIGSR   119
UCP3   57   GVLGTILTMVRTEGPRSPYSGLVAGLHRQMSFASIRIGLYDSVKQFYTPKGADHSSVAIR   116
            ****** *:.:***  . *.** **::**:****:********:::::      :.:. :

UCP1  117   ISAGLMTGGVAVFIGQPTEVVKVRMQAQSHLH-GIKPRYTGTYNAYRVIATTESLSTLWK   175
UCP2  120   LLAGSTTGALAVAVAQPTDVVKVRFQAQARA--GGGRRYQSTVEAYKTIAREEGIRGLWK   177
UCP3  117   ILAGCTTGAMAVTCAQPTDVVKVRFQAMIRLGTGGERKYRGTMDAYRTIAREEGVRGLWK   176
            : **  **.:**  .***:*****:**  :   *   :* .* :**:.**  *.:  ***

UCP1  176   GTTPNLMRNVIINCTELVTYDLMKGALVNNKILADDVPCHLLSALVAGFCTTLLASPVDV   235
UCP2  178   GTSPNVARNAIVNCAELVTYDLIKDTLLKANLMTDDLPCHFTSAFGAGFCTTVIASPVDV   237
UCP3  177   GTWPNITRNAIVNCAEMVTYDIIKEKLLESHLFTDNFPCHFVSAFGAGFCATVVASPVDV   236
            ** **: **.*:**:*:****::*  *:: ::::*:.***: **: ****:*::******

UCP1  236   VKTRFINSLPGQYPSVPSCAMSMYTKEGPTAFFKGFVASFLRLGSWNVIMFVCFEQLKKE   295
UCP2  238   VKTRYMNSALGQYHSAGHCALTMLRKEGPRAFYKGFMPSFLRLGSWNVVMFVTYEQLKRA   297
UCP3  237   VKTRYMNAPLGRYRSPLHCMLKMVAQEGPTAFYKGFVPSFLRLGAWNVMMFVTYEQLKRA   296
            ****::*:  *:* *   * :.*  :*** **:***: ******:***:*** :****:

UCP1  296   LMKSRQTVDCTT                                                   307
UCP2  298   LMAAYQSREAPF                                                   309
UCP3  297   LMKVQVLRESPF                                                   308
            **      :.
```

Figure S5. Alignment of the primary sequences of UCP1, UCP2, and UCP3 proteins from organism *Mus musculus* (mouse). Three conserved arginine residues in the protein cavity, which are relevant for ATP binding in physiological conditions, are shown in red boxes.

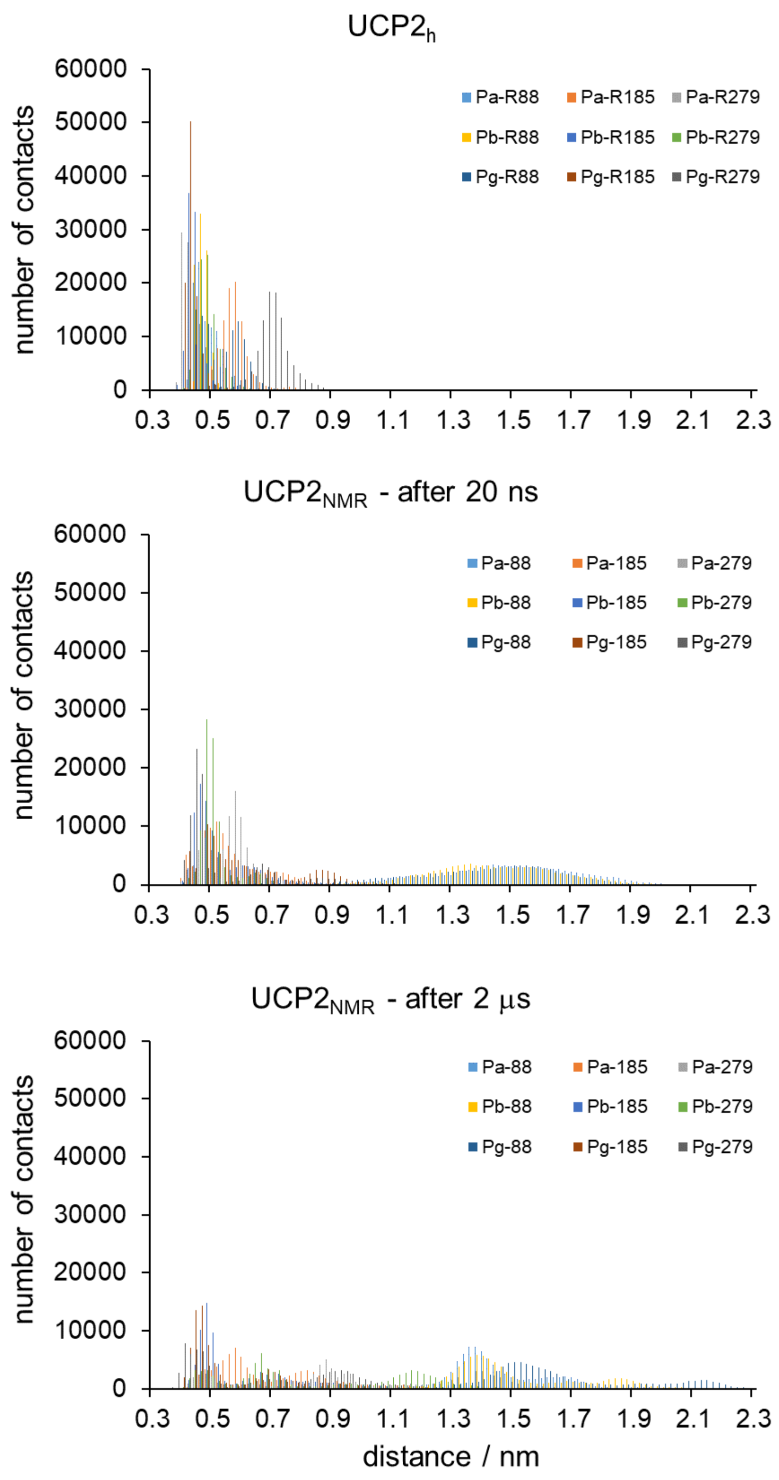

Figure S6. The number of contacts between phosphorous atoms P_{α}, P_{β} and P_{γ} and center of mass of R88, R185 and R279 residues, respectively, vs. the distance between the groups for UCP2$_h$ structure after 2μs (a), UCP2$_{NMR}$ structure after 20 ns (b) and UCP2$_{NMR}$ structure after 2 μs (c).

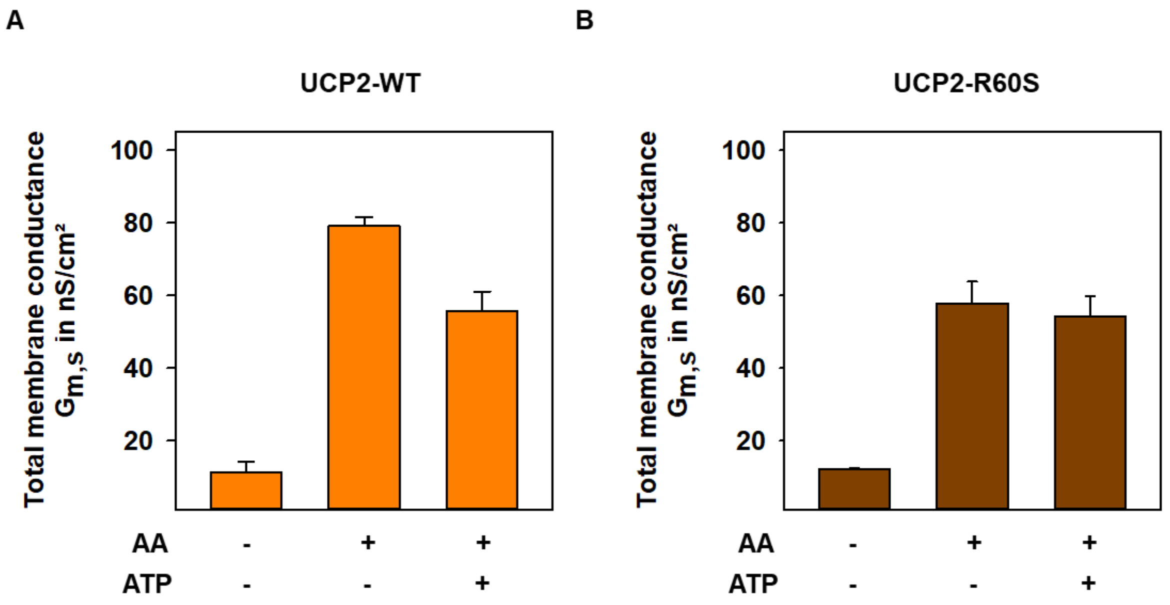

Figure S7. Effect of UCP2-R60S mutant on AA mediated activation in inhibition by ATP. Total membrane specific conductance of UCP2-WT **(A)** and UCP2-R60S **(B)** in the absence of AA (left bars), in the presence of AA (middle bars) and the presence of AA and 2 mM ATP (right bars). In all measurements, buffer contained 50 mM Na_2SO_4,10 mM MES, 10 mM Tris and 0.6 mM EGTA at pH = 7.34 and T = 306 K. Lipid membrane was made of 45:45:10 mol% DOPC:DOPE:CL and 15 mol% AA where indicated. Lipid and protein concentrations were 1.5 mg/mL and 4 µg per mg of lipid. Data are the mean ± SD of three independent experiments.

a) b)

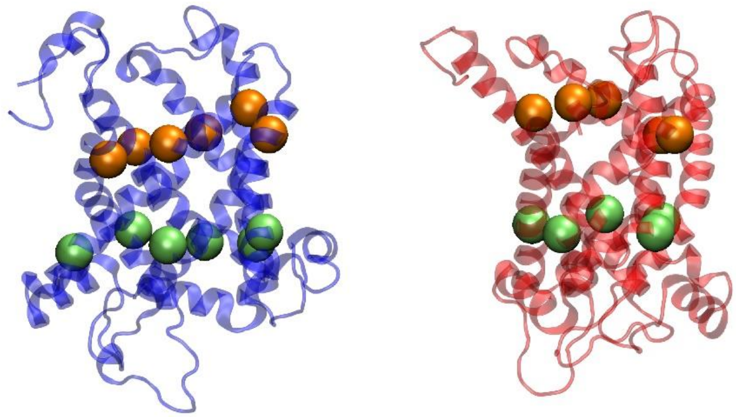

Figure S8. Side view of a) $UCP2_{NMR}$ and b) $UCP2_h$ structures, with C_α atoms used to define cylindrical region necessary for permeability calculations colored orange (top ring) and lime (bottom ring). See the manuscript for further details.

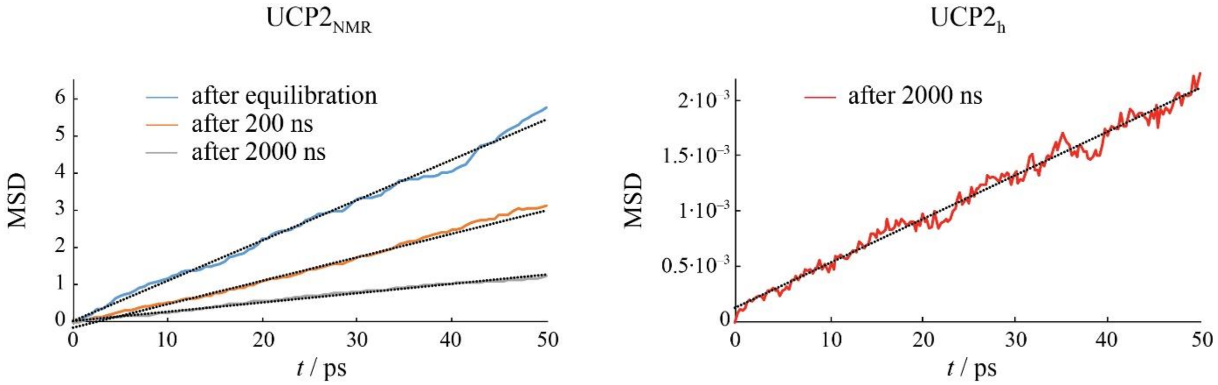

Figure S9. Mean square displacements (MSDs) of collective variable *n* for three $UCP2_{NMR}$ and homologically modeled UCP2 structure ($UCP2_h$). A line with the best-fit slope obtained taking into account region from 10 to 30 ps was superimposed on each MSD curve (black dotted line). The collective diffusion coefficient of water inside the protein, D_n, can be obtained by scaling the slope of the best-fit line by a factor of 0.5 (see manuscript for details). Errors in the calculated D_n values were evaluated by dividing the MSD between 10 to 30 ps into four non-overlapping regions and by recalculating D_n for the obtained subregions. The largest difference from the four obtained subregion D_n values from the D_n value of the entire region is then the error in the obtained D_n.

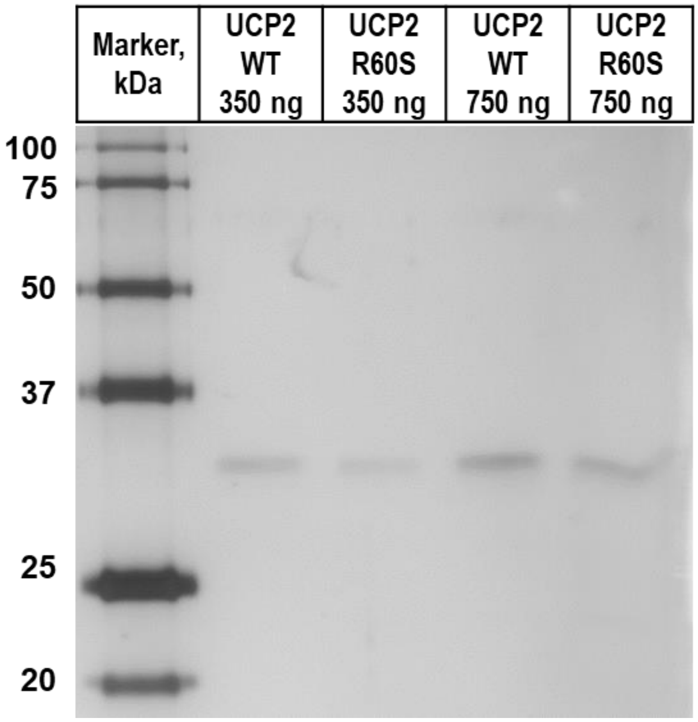

Figure S10. Representative silverstaining of murine UCP2WT and UCP2R60S. For quality control 350 or 750 ng of proteoliposomes were loaded onto a 15% acrylamide gel and SDS-PAGE was performed. Subsequently, proteins were visualized by silver staining. Precision Plus Protein T Dual Color Standard (Bio-Rad) was loaded as a molecular weight marker.

Table S1. Number of water molecules passing through the membrane for $UCP2_h$, $UCP2_{NMR}$ and ANT, where the first 10 ns (0 ns – 10 ns) and 10 ns after 1 µs (1000 ns -1010 ns) of their respective 2 µs free MD simulations were analyzed. Water molecules were added to the "transferred set" only and only if it exchanged the water reservoir by passing through the membrane (matrix side to cytosol side or vice versa) during the analyzed 10 ns of its respective simulation.

t / ns	$UCP2_h$	$UCP2_{NMR}$	ANT
0 – 10	0	41	2
1000 – 1010	0	22	0
